# Thyroid hormones and energy metabolism in amyotrophic lateral sclerosis

**DOI:** 10.1093/braincomms/fcag198

**Published:** 2026-06-04

**Authors:** Liubov Novikova, Christina Lang, Hayrettin Tumani, Veronika Klose, Jan Kassubek, Jens Dreyhaupt, Luc Dupuis, Martin Wabitsch, Albert Ludolph

**Affiliations:** Department of Neurology, Ulm University, Ulm 89081, Germany; Department of Neurology, Ulm University, Ulm 89081, Germany; Department of Neurology, Ulm University, Ulm 89081, Germany; Department of Neurology, German Center of Neurodegenerative Diseases, Ulm 89081, Germany; Department of Neurology, Ulm University, Ulm 89081, Germany; Institute of Epidemiology and Medical Biometry, Ulm 89075, Germany; Université de Strasbourg, Inserm, Strasbourg Translational Neuroscience and Psychiatry, UMR-S1329, Centre de Recherches en Biomédecine, Strasbourg 67000, France; German Center for Child and Adolescent Health (DZKJ), Partner Site Ulm, Division of Pediatric Endocrinology and Diabetes, Department of Pediatrics and Adolescent Medicine, Ulm University, Ulm 89075, Germany; Department of Neurology, Ulm University, Ulm 89081, Germany

**Keywords:** ALS, thyroid function, catabolism, body mass index, neurofilaments

## Abstract

Weight loss, partially caused by hypermetabolism, represents a well-documented and therapeutically relevant feature of the amyotrophic lateral sclerosis phenotype worldwide. In this study, we retrospectively analysed the association between thyroid function and clinical, prognostic and metabolic parameters in a cohort of patients with amyotrophic lateral sclerosis in an experienced centre in Germany (*n* = 1754). Specifically, we examined the relationship between thyroid stimulating hormone levels, age, glucose and body mass index and—in subgroups—phosphorylated neurofilament heavy chain levels in CSF. There was no association between thyroid stimulating hormone levels and body mass index in patients with amyotrophic lateral sclerosis (*n* = 954). In contrast with other cohorts, thyroid stimulating hormone levels decreased with age in patients with amyotrophic lateral sclerosis indicating hypothalamic deficiency in the ageing patients. There was no association between thyroid stimulating hormone and phosphorylated neurofilament heavy chain (prognostic marker) in CSF of a subcohort (*n* = 646). Thyroid stimulating hormone levels correlated with glucose levels, an effect more pronounced in male patients. In conclusion, our results suggest that thyroid metabolism does not significantly contribute to amyotrophic lateral sclerosis-related weight loss or disease prognosis as estimated by phosphorylated neurofilament heavy chain; thyroid dysfunction is unlikely to be a primary driver of the metabolic dysregulation observed in amyotrophic lateral sclerosis. Most interestingly, thyroid stimulating hormone levels show an unexpected negative relation to age in patients with amyotrophic lateral sclerosis.

## Teaser

Weight loss and hypermetabolism are common in amyotrophic lateral sclerosis. Novikova *et al*. found no association between thyroid hormone levels and body mass index or a neurodegenerative prognostic marker. Thyroid function declined with age and correlated with glucose, especially in men, suggesting limited impact on weight loss or prognosis.

## Graphical Abstract

**Graphical Abstract:**
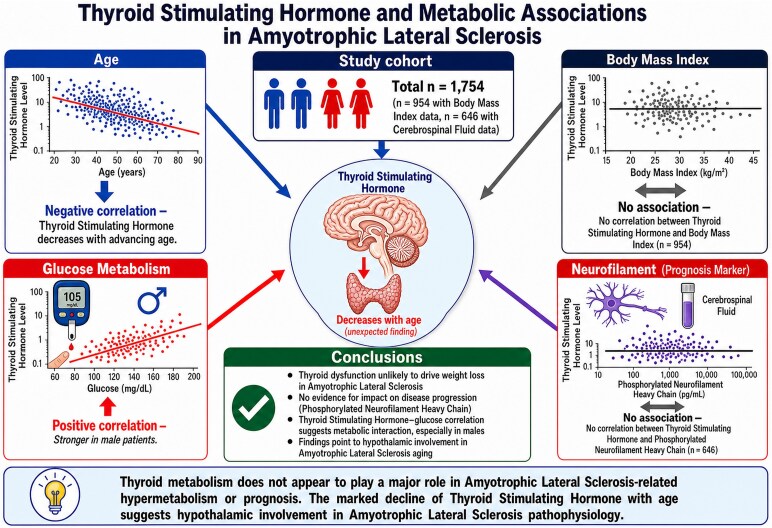

## Introduction

Amyotrophic lateral sclerosis (ALS) is a neurodegenerative disorder with an incidence of up to 3 per 100 000 annually, resulting in 2500 new cases per year in Germany. The disease predominantly affects individuals aged 60–75, with a slight male predominance (55%). Bulbar onset occurs in ∼34% of cases, and the median survival time is 31 months. It is estimated that by 2050 the incidence of ALS in Europe will increase to 4.5 per 100 000 in men and 3.3 per 100 000 in women, mainly because of demographic change.^1-3^

Traditionally, ALS has been associated with selective motor neuron degeneration; however, recent evidence shows that it is rather a multisystem disorder extending beyond the motor system to include significant behavioural, cognitive and metabolic alterations.^4-8^ These systemic manifestations emphasize that neurobiological dysfunction extends beyond motor neuron loss.

A hallmark feature of ALS is catabolism, which already plays a role in the preclinical phases of the disease: before the onset of clinical symptoms, ALS patients exhibit metabolic changes, such as increased energy expenditure and significant weight loss, reflecting dysregulated metabolism that may accelerate disease progression. Independent studies^6,9-11^ demonstrated that a lack of an increase of the body mass index (BMI) in midlife differentiates preclinical ALS patients from controls; also, a lower BMI correlates with a poorer prognosis.^6^ Additionally, metabolic abnormalities like dyslipidaemia and glucose intolerance have been documented both in preclinical and advanced stages of ALS.^12-14^

These findings suggest a critical involvement of systemic metabolism in ALS progression. A potential contributor to this dysregulation is thyroid dysfunction, which has long been suspected as a probable pathogenetic factor in ALS,^15^ although conclusive evidence is lacking. Thyroid metabolism plays a central role in regulating systemic energy metabolism. Thyroid hormones increase resting energy expenditure (REE), promote the breakdown of fats and carbohydrates and regulate protein homeostasis, impacting insulin sensitivity and glucose uptake in skeletal muscle, liver and adipose tissue.^16,17^ Dysregulation of thyroid function could thus contribute to the metabolic changes seen in ALS, particularly in the preclinical phase where morphological and functional alterations of the hypothalamus are present.^18-21^

Recent research on the role of neurofilaments (Nf) in ALS linked increased levels of neurofilament light (NfL) and heavy (NfH) chains to motor neuron degeneration and has identified these biomarkers as indicators of disease progression.^7,22^ Notably, lower BMI was associated with higher Nf levels, further suggesting that nutritional status and muscle mass are factors influencing ALS progression. The relationship between thyroid function and the axonal marker Nfs is not explored.

The aim of this study was to analyse the potential relationship between thyroid function and clinical parameters (specifically BMI), age and the progression marker phosphorylated neurofilament heavy chain (pNfH) in patients with ALS.

## Materials and methods

This study was performed as a cross-sectional retrospective data analysis conducted at the Department of Neurology, University Hospital of Ulm. Patient identification was performed using the ICD-10 code G12.21 for ALS in the clinical documentation system MCC, covering the period from 2010 to 2024.

### Study cohort

A total of 1754 patients with a confirmed definite diagnosis of ALS according to the El Escorial criteria were included in the study cohort, based on predefined inclusion and exclusion criteria (see below). These individuals were enrolled in a prospectively maintained hospital-based ALS registry, established in 2010, which ensures systematic and longitudinal recording of clinical, laboratory and demographic variables. In parallel, quantification of CSF levels of pNfH was performed using a standardized enzyme-linked immunosorbent assay protocol.^7^ Diagnostic confirmation according to the El Escorial criteria was supported by laboratory biomarker findings. By March 2024, a sufficient number of cases had been recruited to ensure meaningful results and thus the feasibility of the project.

To eliminate potential confounding from thyroid replacement therapy, patients receiving L-thyroxine were excluded from the main analysis, and only those without L-thyroxine treatment were included in the subsequent subanalysis.

### Methodology

Standardized data collection systematically included demographic information (age, sex, genetic history, clinical phenotype), clinical-neurological findings (anthropometric parameters measured using the BMI formula) and laboratory values (routine venous blood samples as part of the diagnostic workup: thyroid function tests [thyroid stimulating hormone (TSH), free triiodothyronine (fT3), free thyroxine (fT4)] and glucose (venous, not fasting) and neurodegenerative markers (pNfH in CSF). The measurement of TSH, as well as fT3 and fT4, was performed in the same laboratory using standard serum immunoassays. TSH levels were routinely measured as part of the diagnostic protocol for ALS and were available for all patients. In contrast, fT3 and fT4 were assessed additionally in a smaller subset of patients. These levels were also analysed in relation to the timing of the initial ALS diagnosis. Blood samples were obtained in the morning, typically prior to breakfast. However, neither the exact timing of collection nor the duration of pre-sampling fasting was standardized, which may have introduced variability in metabolic parameters (see below). BMI measurements and other variables were obtained at the time of diagnosis, typically within 1 week of hospital admission.

### Inclusion criteria

- Age between 16 and 95 years
- Confirmed definite ALS diagnosis according to the El Escorial criteria
- Availability of routine laboratory parameters (including thyroid function) as well as BMI data

### Exclusion criteria

- TSH values outside the range of 0–10 mU/L
- BMI values < 10 or >45 kg/m^2^

### Data handling and ethical considerations

All patient data were pseudonymized after collection, and analyses were conducted on anonymized data to ensure confidentiality. The study adhered to the principles of the Declaration of Helsinki and was approved by the Ethics Commission of the University of Ulm (N30/2025). Due to the retrospective nature of the analysis, all data were handled in compliance with ethical standards and data protection regulations.

### Statistical analysis

As part of this research project, an exploratory data analysis was conducted. Qualitative variables were summarized using absolute and relative frequencies, while quantitative variables were characterized by the mean, median, minimum, maximum, lower quartile (Q1), upper quartile (Q3) and standard deviation (SD). All patients were comprehensively characterized, including demographic, clinical and laboratory parameters, stratified by sex and, where appropriate, complemented by graphical representations. Differences in demographic, clinical and laboratory parameters between men and women were assessed using the Mann–Whitney U-test.

Relationships between thyroid parameters, clinical-neurological and laboratory variables in the overall cohort were initially evaluated using bivariate scatterplots and Spearman’s partial rank correlation coefficients, controlling for sex as a confounding factor. Two-sided *P*-values < 0.05 were considered statistically significant. In addition to these standard analyses, all correlation *P*-values were further adjusted for multiple comparisons using the Bonferroni correction to control the risk of type I errors. Statistical analysis was done using program ‘Statistica 12’, Version 13.5.0.17.

## Results

The present retrospective analysis comprised 1757 patients with a definite diagnosis of ALS. Three individuals were excluded due to TSH concentrations > 10 mU/L. Additionally, 60 patients exhibited TSH levels outside the reference range (0.2–3.4 mU/L) but were retained in the analysis. The final study cohort therefore included 1754 patients (693 females and 1061 males) with a median age of 63.81 (55.74; 71.49) years. No cases of clinically manifested Hashimoto’s thyroiditis, remitted Graves’ disease or benign nodular thyroid disease were identified among the 1754 patients included in the study. Table 1 summarizes the clinical characteristics of the ALS cohort. The sex distribution revealed a predominance of males (60.5%, *n* = 1061) compared to females (39.5%, *n* = 693), with a male-to-female ratio of ∼1.5:1. Family history was positive for ALS and/or frontotemporal dementia (FTD) in 157 patients (9.0%), corresponding to 10.5% of females and 7.9% of males. The vast majority, 1576 patients (89.8%), had a negative family history, while in 21 cases (1.2%) information was inconclusive or unavailable. Four hundred and seventy-eight patients (27.3%) presented with a bulbar onset, which was markedly more frequent in women (37.7%) than in men (20.5%). Conversely, 1276 patients (72.7%) exhibited spinal onset, predominating in males (79.5%) compared to females (62.3%). The characteristics of this cohort (slight male predominance, higher frequency of bulbar onset in women and predominance of spinal onset in men) reflect established sex-specific clinical patterns of the disease.^2,23,24^

**Table 1:** Clinical characteristics of the ALS cohort

Characteristic	Total *N* (%)	Females *N* (%)	Males *N* (%)
Sex distribution	1754 (100%)	693 (39.5%)	1061 (60.5%)
Family history			
Positive	157 (9.0%)	73 (10.5%)	84 (7.9%)
Negative	1576 (89.8%)	612 (88.3%)	964 (90.9%)
Unknown	21 (1.2%)	8 (1.2%)	13 (1.2%)
Clinical manifestation			
Bulbar onset	478 (27.3%)	261 (37.7%)	217 (20.5%)
Spinal onset	1276 (72.7%)	432 (62.3%)	844 (79.5%)

Clinical and metabolic parameters of the cohort are presented in Table 2. Thyroid function was evaluated indirectly via measurement of serum TSH, with a median level of 1.01 (0.68; 1.45) mU/L, indicating overall euthyroid status within the cohort. FT3 and fT4 levels, measured in smaller subsets of patients (*n* = 56 and *n* = 63, respectively), had median values of 2.76 (2.54; 3.14) pg/mL and 1.05 (0.97; 1.24) ng/dL, both within standard reference ranges. Metabolic parameters were further assessed with median glucose concentrations of 5.92 (5.23; 7.14) mmol/L, measured in 1731 patients. The neurodegenerative axonal marker pNfH in CSF showed a median concentration of 1924.00 (879.00; 3537.00) pg/mL, based on data from 646 patients, reflecting substantial interindividual variability. BMI was available for 954 patients, with a median of 24.49 (22.16; 27.64) kg/m^2^, suggesting that the majority of patients were within the normal to overweight range.

**Table 2:** Descriptive statistics of demographic, biochemical and clinical variables in the ALS cohort

	Valid *N*	Mean	Median	Minimum	Maximum	Lower quartile	Upper quartile	Std. dev.
Age	1754.00	63.07	63.81	16.25	93.16	55.74	71.49	11.48
TSH	1754.00	1.17	1.01	0.01	9.54	0.68	1.45	0.80
FT3	56.00	2.77	2.76	1.68	3.45	2.54	3.14	0.41
FT4	63.00	1.12	1.05	0.75	2.23	0.97	1.24	0.23
Glucose	1731.00	6.43	5.92	3.19	25.95	5.23	7.14	1.89
pNfH_CSF	646.00	2551.93	1924.00	188.00	16 459.00	879.00	3537.00	2323.36
BMI	954.00	25.03	24.49	13.13	44.96	22.16	27.64	4.28

A comparative analysis was performed to investigate potential sex-related differences in clinical and laboratory parameters, aiming to provide a better understanding of how sex may influence disease manifestation and progression. As summarized in Supplementary Table 1, women were slightly older on median age than men (64.63 (56.99; 71.99) versus 63.20 (54.90; 71.32) years; *P* = 0.0221) and exhibited a lower BMI (24.13 (21.45; 27.48) versus 24.68 (22.60; 27.72) kg/m^2^; *P* = 0.0133). Analysis of hormonal parameters revealed no significant sex differences in TSH levels [0.99 (0.64; 1.46) versus 1.02 (0.70; 1.44) mU/L; *P* = 0.2393]. Similarly, glucose concentrations were comparable between women and men [5.89 (5.24; 7.04) versus 5.96 (5.23; 7.20) mmol/L; *P* = 0.3552]. Regarding neurodegenerative biomarkers, there was no statistically significant difference in CSF pNfH levels between males and females [1899.00 (786.00; 3546.00) pg/mL versus 1965.00 (1041.00; 3363.00) pg/mL; *P* = 0.4857]. Overall, these findings indicate modest sex-related differences in age and BMI, whereas hormonal and neurodegenerative markers were largely similar between sexes.

From the entire cohort, male and female participants with available data on age, TSH, glucose and BMI were selected. This study cohort consisted of 940 participants. The median age was 63.93 (55.90; 71.33) years. Median TSH levels were 1.01 (0.68; 1.46) mU/L, indicating largely normal thyroid function within the cohort. Median glucose levels were 5.99 (5.23; 7.08) mmol/L, and median BMI was 24.49 (22.18; 27.65) kg/m^2^, representing a range from underweight to obese. The data were generally comparable to the entire cohort (Supplementary Table 2).

The correlation analysis presented in Table 3 shows that age was weakly negatively correlated with TSH (*r* = −0.0920, *P* < 0.05) and BMI (*r* = −0.1017, *P* < 0.05) and positively correlated with glucose (*r* = 0.1501, *P* < 0.05). These associations remained statistically significant after Bonferroni correction (§). TSH and glucose were negatively correlated (*r* = −0.1051, *P* < 0.05), an association that also remained significant following Bonferroni adjustment. BMI showed a modest positive correlation with glucose (*r* = 0.0869, *P* < 0.05) at the unadjusted *P*-value level and after Bonferroni adjustment (scatterplots in Supplementary Figs 1–5). Additional analyses were conducted across the cohort, stratified by sex, to evaluate potential differences between males and females in the correlations among demographic, laboratory and clinical parameters. Among females from this cohort (*n* = 369; median age 64.91 (56.52; 71.86) years; Supplementary Table 3), after Bonferroni adjustment, only a positive correlation between age and glucose was observed (*r* = 0.1649, *P* < 0.05). No other correlations reached statistical significance after correction, including those between TSH and the other variables, indicating limited monotonic associations in this subgroup (Supplementary Table 4). Scatterplot of the significant correlations across the female patients are presented in Supplementary Fig. 6.

**Table 3:** Correlation between demographic, biochemical and clinical parameters

	Age	TSH	Glucose	BMI
Age	1.0000	**−0.0920** ^a,b^	**0.1501** ^a,b^	**−0.1017** ^a,b^
TSH	**−0.0920** ^a,b^	1.0000	**−0.1051** ^a,b^	0.0365
Glucose	**0.1501** ^a,b^	**−0.1051** ^a,b^	1.0000	**0.0869** ^a,b^
BMI	**−0.1017** ^a,b^	0.0365	**0.0869** ^a,b^	1.0000

^a^ *P* < 0.05 based on Spearman rank-order correlations.

^b^Significance after Bonferroni adjustment.

Bold values indicate statistically significant Spearman rank-order correlations (*P* < 0.05).

Male patients [*n* = 571, median age 63.36 (55.25; 71.12) years; descriptive statistics presented in Supplementary Table 5] exhibited a weak negative correlation between age and TSH (*r* = −0.1210, *P* < 0.05), as well as between age and BMI (*r* = −0.1159, *P* < 0.05), indicating decreases of these parameters with age. Conversely, age positively correlated with blood glucose (*r* = 0.1434, *P* < 0.05); TSH negatively correlated with glucose (*r* = −0.1506, *P* < 0.05) (Supplementary Table 6). Scatterplots of the significant correlations across the male patients are presented in Supplementary Figs 7–10.

While the correlations between age, TSH, glucose and BMI provide insights into potential metabolic influences on the metabolic status of ALS patients, they do not adequately reflect the dynamics of disease progression due to neurodegeneration. To address this limitation and to explore whether thyroid or metabolic parameters might be linked to the underlying neurodegenerative process, an additional analysis was conducted incorporating the pNfH as a biomarker. For this analysis, the required data of *age, TSH, glucose, BMI and pNfH in CSF* were available for 340 patients [118 females and 222 males, median age 63.23 (55.20; 70.27) years]. This cohort is presented in Supplementary Table 7. As shown in Table 4, only weak correlations were observed across the variables. Age demonstrated a weak positive correlation with glucose levels (*r* = 0.2435, *P* < 0.05), which remained statistically significant after Bonferroni adjustment (§). Conversely, weak negative correlations were found between TSH and both age (*r* = −0.1088, *P* < 0.05) and glucose (*r* = −0.1181, *P* < 0.05). No significant associations between pNfH and metabolic or endocrine markers (TSH, glucose, BMI) were detected, indicating that pNfH concentrations, as a neurodegenerative biomarker, reflect neuroaxonal damage independently of peripheral metabolic alterations.

**Table 4:** Correlation between demographic, biochemical and clinical parameters among 340 patients

	Age	TSH	Glucose	pNfH_CSF	BMI
Age	1.0000	**−0.1088** ^a^	**0.2435** ^a,b^	−0.1019	−0.0583
TSH	**−0.1088** ^a^	1.0000	**−0.1181** ^a^	0.0846	0.0004
Glucose	**0.2435** ^a,b^	**−0.1181** ^a^	1.0000	−0.0198	0.0956
pNfH_CSF	−0.1019	0.0846	−0.0198	1.0000	−0.0793
BMI	−0.0583	0.0004	0.0956	−0.0793	1.0000

^a^ *P* < 0.05 based on Spearman rank-order correlations.

^b^Significance after Bonferroni adjustment.

Bold values indicate statistically significant Spearman rank-order correlations (*P* < 0.05).

A subsequent sex-specific correlation analysis was conducted to investigate potential associations between endocrine, metabolic and neurodegenerative parameters within each subgroup. Among female patients [*n* = 118; median age 63.09 (55.45; 70.89) years; descriptive statistics in Supplementary Table 8], no significant correlations were observed between the analysed parameters (Supplementary Table 9). In male patients [*n* = 222; median age 63.23 (55.05; 70.06) years; descriptive statistics in Supplementary Table 10], the correlation pattern largely reflected that of the overall cohort. Glucose levels were positively correlated with age (*r* = 0.2902, *P* < 0.05) and negatively correlated with TSH (*r* = −0.1955, *P* < 0.05). No significant correlations were found between the neurodegenerative marker pNfH and any of the studied parameters (Supplementary Table 11).

In the entire ALS cohort (*n* = 940), as well as in the analysed subcohort (*n* = 340), persistent negative correlations were observed between TSH and both age and glucose, alongside a positive correlation between age and glucose, reflecting subtle age-related changes in metabolism and thyroid function. Sex-specific analyses revealed distinct patterns: in males, age was modestly associated with higher glucose and lower TSH and BMI, suggesting more pronounced metabolic and endocrine shifts with age, whereas in females, only a weak positive correlation between age and glucose was observed, with no significant associations for TSH. Importantly, the neurodegenerative biomarker pNfH in CSF showed no significant correlations with demographic, biochemical and clinical parameters across the whole cohort and within each sex, indicating that neuroaxonal degeneration occurs independently of peripheral metabolic or endocrine status. While metabolic and endocrine parameters display subtle sex-specific patterns, these findings suggest that they do not directly influence the severity of neurodegeneration as reflected by pNfH in CSF.

To avoid the influence of thyroid supplementation therapy, patients receiving L-thyroxine were excluded from the analysis. A total of 234 patients (82 males, 152 females) were receiving substitution therapy with L-thyroxine. To control for potential confounding effects of exogenous thyroid hormone administration on thyroid function parameters, correlation analyses were repeated after exclusion of these individuals from the overall cohort.

Only individuals with available clinical and biochemical parameters with the normal thyroid function were included, resulting in a cohort of 831 participants [median age 63.47 (55.38; 71.76) years]. Descriptive statistics for demographic, biochemical and clinical variables are presented in Supplementary Table 12, representing the overall cohort’s characteristics. The correlation analysis in this cohort demonstrated a statistically significant negative relationship between age and TSH levels (*r* = −0.1281, *P* < 0.05), while no substantial correlation was found between TSH and BMI (Table 5).

**Table 5:** Correlation between demographic, biochemical and clinical parameters of patients without L-thyroxine substitution therapy

	Age	TSH	Glucose	BMI	pNfH_CSF
Age	1.0000	**−0.1281** ^a^	0.0068	0.0432	0.0621
TSH	**−0.1281** ^a^	1.0000	−0.0062	−0.0304	−0.0189
Glucose	0.0068	−0.0062	1.0000	0.0147	0.0890
BMI	0.0432	−0.0304	0.0147	1.0000	0.0271
pNfH_CSF	0.0621	−0.0189	0.0890	0.0271	1.0000

^a^ *P* < 0.05 based on Spearman rank-order correlations.

Bold values indicate statistically significant Spearman rank-order correlations (*P* < 0.05).

Sex-specific analyses were conducted for females [*n* = 305, median age 62.93 (55.62; 71.32) years, with descriptive statistics presented in Supplementary Table 13 and correlation data in Table 6] and males [*n* = 526, median age 63.60 (55.34; 71.79) years, with descriptive statistics in Supplementary Table 14 and correlation data in Table 7]. These analyses revealed no statistically significant correlation between BMI and thyroid function in either group. The negative correlation between age and TSH (*r* = −0.2334, *P* < 0.05) observed in this cohort was consistent with the overall cohort findings.

**Table 6:** Correlation between demographic, biochemical and clinical parameters among female patients without L-thyroxine substitution therapy

	Age	TSH	Glucose	BMI	pNfH_CSF
Age	1.0000	**−0.2334** ^a^	−0.0502	0.0162	0.0621
TSH	**−0.2334** ^a^	1.0000	0.0072	0.0067	−0.0189
Glucose	−0.0502	0.0072	1.0000	−0.0335	0.0890
BMI	0.0162	0.0067	−0.0335	1.0000	0.0271
pNfH_CSF	0.0621	−0.0189	0.0890	0.0271	1.0000

^a^ *P* < 0.05 based on Spearman rank-order correlations.

Bold values indicate statistically significant Spearman rank-order correlations (*P* < 0.05).

**Table 7:** Correlation between demographic, biochemical and clinical parameters among male patients without L-thyroxine substitution therapy

	Age	TSH	Glucose	BMI	pNfH_CSF
Age	1.0000	−0.0697	0.0415	0.0599	0.0430
TSH	−0.0697	1.0000	−0.0177	−0.0563	0.0129
Glucose	0.0415	−0.0177	1.0000	0.0376	0.1293
BMI	0.0599	−0.0563	0.0376	1.0000	0.0326
pNfH_CSF	0.0430	0.0129	0.1293	0.0326	1.0000

In the cohort of patients with normal thyroid function and without L-thyroxine therapy, TSH levels exhibited a similar trend, showing a statistically significant negative correlation with age. However, no meaningful correlation was observed between TSH and BMI, either in the overall cohort or within sex-specific subgroups. These findings suggest that, in individuals with intact thyroid function, thyroid status does not appear to be a major determinant of BMI or the catabolic state in patients with ALS.

## Discussion

This retrospective analysis of 1754 ALS patients included a slightly higher proportion of males (60.5%) compared to females (39.5%), with the median age of 63.81 (55.74; 71.49) years, consistent with previous studies showing a higher incidence of ALS in men.^3,23,25,26^ A family history of ALS was documented in 9% of patients. 27.3% had bulbar, while 72.7% had spinal onset, also consistent with findings from other ALS studies.^1,3,23,27^

### Normal thyroid function in amyotrophic lateral sclerosis

Thyroid function remained within normal limits [median: 1.01 (0.68; 1.45) mU/L] in the cohort, with no significant differences between males and females and no clinically relevant deviations. According to our inclusion criteria, we excluded three patients with TSH levels > 10 mU/L and 60 patients had a TSH outside the reference range (0.2–3.4 mU/L). FT3 and fT4 levels, measured in smaller subsets of patients, were both within standard reference ranges. These results confirm previous studies that did not show an increased prevalence of thyroid dysfunction in ALS.^28,29^ Although Zheng *et al.*^15^ reported a link between fT3 levels and survival, this association disappeared after adjusting for confounders, suggesting a secondary role.

Although glucose was measured in a non-fasting state, the median level [5.92 (5.23; 7.14) mmol/L] did not differ between sexes. Non-fasted glucose values provide limited information unless clearly elevated. Nevertheless, previous studies, such as Pradat *et al.*,^30^ have reported impaired glucose metabolism in ALS, linking glucose intolerance to elevated free fatty acids and insulin resistance. The absence of sex differences in our cohort supports a disease-related rather than sex-specific mechanism.

Across the cohort, a positive correlation was observed between age and glucose levels among the patients with ALS. This trend was observed in both males and females, although among females this correlation was slightly stronger. Ageing is known to impair glucose metabolism associated with glucose intolerance and insulin resistance.^31,32^ These findings suggest that, similar to the general ageing population, ALS patients may also experience age-related metabolic dysfunction, potentially exacerbated by the progression of the disease. This highlights the importance of monitoring glucose levels and metabolic function in ALS patients, particularly with advancing age.

### Correlation of thyroid function with metabolic, clinical and neurodegenerative parameters in amyotrophic lateral sclerosis

In clinical endocrinology, serum TSH remains the primary, most sensitive and cost-effective biomarker for the initial evaluation and longitudinal monitoring of thyroid function. Nevertheless, free T4 and free T3 levels should also be carefully considered—particularly in the setting of synthetic hormone replacement or equivocal biochemical profiles—since comprehensive assessment requires their integration, along with exclusion of analytical interferents such as macro-TSH, to ensure diagnostic accuracy and therapeutic precision.^31,32^

In our cohort, thyroid function was within normal range, as evidenced by serum TSH, fT3 and fT4 concentrations. Nevertheless, while TSH levels in our cohort remained normal, they appeared lower than in longitudinal and population-based studies, which consistently demonstrate a progressive rise in serum TSH concentrations with advancing age, including individuals free from overt thyroid disease. The Busselton Health Survey, which followed over 1100 participants for 13 years, reported a mean TSH increase from 1.49 to 1.81 mU/L, while free T4 remained stable indicating a physiological age-related shift rather than early thyroid failure.^33,34^

Our study revealed a significant negative correlation between TSH levels and age in ALS patients, particularly in males (*r* = −0.1210, *P* < 0.05), suggesting a sex-dependent modulation of thyroid function. This finding contrasts with the typical age-related rise in TSH observed in control populations, where the hypothalamic-pituitary-thyroid (HPT) axis shows reduced responsiveness with age.^35,36^ Remarkably, we did not observe the expected increase in TSH with age, as seen in both normal and disease-related cohorts.^37-41^

We hypothesized that this deviation may reflect underlying neuroendocrine alterations in ALS, for example, the previously described hypothalamic atrophy^19^ and the MCH/orexin imbalance,^18,21^ which could impair the regulation of the HPT axis. Similarly, the lack of an age-related increase in the albumin quotient, a marker of blood-CSF barrier function, in ALS patients^42^ may suggest broader disruptions in neurovascular and endocrine adaptations to ageing in ALS.

We observed a weak but statistically significant negative correlation between TSH and glucose levels (*r* = −0.1051, *P* < 0.05), more pronounced in males (*r* = −0.1506, *P* < 0.05), which may suggest a possible sex-related interaction between thyroid function and glucose metabolism. However, given the small effect sizes and predominantly normal TSH values, this finding should be interpreted as exploratory rather than indicative of clinically relevant thyroid dysfunction. This association may reflect potential hypothyroidism-induced hypoglycaemic effects, in contrast to prior studies showing that hypothyroidism impairs glucose uptake and worsens insulin resistance.^43,44^ While Kalra *et al.*^45^ highlighted hyperthyroidism-induced hyperglycaemia, our findings imply a distinct metabolic profile in ALS. These discrepancies underscore the complexity of thyroid-glucose interplay in ALS, potentially influenced by neurodegeneration, metabolic dysregulation, muscle loss and sex-specific hormonal modulation. Importantly, TSH levels showed no significant correlation with BMI, neither in the overall cohort nor within sex subgroups. To minimize potential confounding, patients receiving supplemental L-thyroxine therapy were excluded from a subgroup analysis. This analysis revealed no significant association between TSH and BMI, suggesting that thyroid function does not directly influence body mass in this cohort. Previous studies in euthyroid individuals suggest a positive association between TSH and BMI.^46,47^ This supports the interpretation that thyroid metabolism is not a causal factor underlying catabolism in ALS. This is also complementary to the finding in a subgroup of more than 600 ALS patients, in which no significant association was found between TSH levels and the prognostic marker CSF pNfH concentrations.

### Sex-dependent correlations among patients with amyotrophic lateral sclerosis

A positive correlation between glucose and BMI suggests persistent metabolic dysregulation in ALS. A tendency to a more pronounced correlation was observed in women, possibly due to hormonal influences such as oestrogen decline.^48^ While some studies suggest hyperglycaemia might be protective in ALS, the metabolic role of adiposity remains complex.^49,50^ With regard to *sex differences*, both female and male patients exhibited a positive correlation between age and glucose levels, indicating that both groups experience similar metabolic and functional challenges with ageing. However, the degree and nature of these correlations varied between sexes.

However, the correlation coefficients observed in this analysis are relatively modest, suggesting that any associations between the examined variables are likely to be weak. Due to the large sample size, even small effects can reach statistical significance. Therefore, it is important to interpret these findings with appropriate caution, as the observed correlations may reflect subtle or complex relationships that are not fully captured by simple correlation analysis. While these results should not be dismissed, they should be considered as part of a broader context, acknowledging that weak correlations may still reveal meaningful insights. To gain a clearer understanding of these associations, further studies utilizing complementary methodologies, such as longitudinal or mechanistic approaches, would be beneficial to better elucidate the underlying factors involved.

### Limitations of the study

We acknowledge several limitations of our study. First, blood glucose levels were measured in the morning prior to breakfast without a standardized fasting period. While this timing reduces postprandial variability, the absence of a defined fasting interval may have introduced some degree of variation in glucose values. Fasting glucose measurements, obtained under controlled metabolic conditions, are generally more robust indicators of glucose homeostasis and should be prioritized in future studies to enhance comparability and interpretability. Another limitation of our study concerns the potential variability in TSH levels due to their known circadian rhythm. TSH exhibits diurnal fluctuations, typically peaking during the night and reaching its lowest levels in the late morning. In our cohort, TSH measurements were performed as part of the standard admission laboratory panel, usually in the early morning hours on the day of hospital admission. However, as the exact timing was not standardized across all patients, this physiological variability may have contributed to interindividual differences in TSH levels and introduced measurement noise. Future studies should consider time-controlled sampling protocols to minimize this source of variability and improve comparability. Finally, it was a cross-sectional study design, which does not allow assessment of longitudinal changes in TSH. Further studies are needed to determine the temporal dynamics of TSH regulation and the mechanisms underlying thyroid function in ALS.

In addition to BMI and TSH, fT3 and fT4 as well as lipid profile were assessed in only a small subset of patients. Due to the limited sample size and incomplete data, this subgroup was underpowered for robust statistical analysis and was excluded from the final evaluation. Consequently, our conclusions are primarily based on BMI and TSH, with limited supporting information from lipid parameters.

Future studies should include a broader panel of metabolic and endocrine markers, such as comprehensive lipid profiling and additional hypothalamic hormonal axis, to more fully characterize systemic metabolic alterations in ALS.

## Conclusions

In conclusion, this study offers novel insights into the interplay between thyroid function and metabolic alterations in a large cohort of patients with ALS. Most importantly, although thyroid function remained within normal limits, a negative correlation between TSH levels and age in the whole cohort may suggest hypothalamic deficiency in the ageing ALS patient. This is consistent with the apparent lack of a response of the hypothalamus to pioglitazone,^51^ a decreased volume of the hypothalamus^19^ and an imbalance of the MCH/orexin axis in ALS patients.^21^ Secondly, the absence of a significant correlation between TSH and BMI indicates that thyroid dysfunction is unlikely to play a major role for the ALS phenotype. Further research is needed to explore the genetic and metabolic causes for catabolism, a prognostic factor in ALS. These results of this study showed that metabolic effects of the thyroid axis are unlikely to make a major contribution but are a framework for further studies.

## Supplementary Material

fcag198_Supplementary_Data

## Data Availability

The data supporting the findings of this study are available from the corresponding author upon reasonable request. Due to patient confidentiality and ethical restrictions, individual-level clinical data cannot be made publicly available. No custom code or mathematical algorithms were generated during the current study.
