# Supplementary material for: Thyroid hormones and energy metabolism in amyotrophic lateral sclerosis
Source: Brain Commun. 2026 Jun 4;8(3):fcag198. doi: 10.1093/braincomms/fcag198 (PMC13256926; doi:10.1093/braincomms/fcag198)
Supplement: fcag198_Supplementary_Data [file fcag198_supplementary_data.pdf]

Supplementary material

Supplementary Table 1 Comparative gender analysis of demographic, biochemical and clinical parameters

	Sex	Valid N	Mean	Median	Minimum	Maximum	Lower Quartile	Upper Quartile	Std.Dev.	*P*-value
Age	males	1,061	62.52	63.20	16.25	88.77	54.90	71.32	11.86	**0.0221***
	females	693	63.89	64.63	19.21	93.16	56.99	71.99	10.84	
TSH	males	1,061	1.16	1.02	0.01	9.54	0.70	1.44	0.71	0.2393
	females	693	1.19	0.99	0.01	9.51	0.64	1.46	0.92	
Glucose	males	1053	6.48	5.96	3.26	25.95	5.23	7.20	1.96	0.3552
	females	678	6.35	5.89	3.19	20.48	5.24	7.04	1.80	
pNfH_CSF	males	409	2,579.39	1,899.00	188.00	16,459.00	786.00	3,546.00	2,489.79	0.4857
	females	237	2,504.55	1,965.00	188.00	10,078.00	1,041.00	3,363.00	2,008.11	
BMI	males	575	25.19	24.68	13.13	43.21	22.60	27.72	3.91	**0.0133***
	females	379	24.78	24.13	14.27	44.96	21.45	27.48	4.78	

*$P < 0.05$ as determined by the Mann-Whitney U test.

Supplementary Table 2 Descriptive statistics of demographic, biochemical and clinical variables in the ALS cohort

	Valid N	Mean	Median	Minimum	Maximum	Lower Quartile	Upper Quartile	Std.Dev.
Age	940.00	63.24	63.93	23.89	88.77	55.90	71.33	11.18
TSH	940.00	1.18	1.01	0.01	9.54	0.68	1.46	0.82
Glucose	940.00	6.46	5.99	3.19	20.48	5.23	7.08	1.92
BMI	940.00	25.03	24.49	13.13	44.96	22.18	27.65	4.27

Supplementary Table 3 Descriptive statistics of demographic, biochemical and clinical variables in the ALS cohort (females)

	Valid N	Mean	Median	Minimum	Maximum	Lower Quartile	Upper Quartile	Std.Dev.
Age	369.00	63.95	64.91	25.14	87.84	56.52	71.86	10.52
TSH	369.00	1.19	1.00	0.01	9.51	0.64	1.42	0.93
Glucose	369.00	6.42	5.96	3.19	20.48	5.24	7.04	1.99
BMI	369.00	24.77	24.21	14.27	44.96	21.45	27.48	4.76

Supplementary Table 4 Correlation between demographic, biochemical and clinical parameters among females

	Age	TSH	Glucose	BMI
Age	1.0000	-0.0856	**0.1649*§**	-0.0716
TSH	-0.0856	1.0000	-0.0442	-0.0104
Glucose	**0.1649*§**	-0.0442	1.0000	0.0973
BMI	-0.0716	-0.0104	0.0973	1.0000

*$P < 0.05$ based on Spearman rank-order correlations
§ denote significance after Bonferroni adjustment

Supplementary Table 5 Descriptive statistics of demographic, biochemical and clinical variables in the ALS cohort (males)

	Valid N	Mean	Median	Minimum	Maximum	Lower Quartile	Upper Quartile	Std.Dev.
Age	571.00	62.78	63.36	23.89	88.77	55.25	71.12	11.58
TSH	571.00	1.18	1.02	0.04	9.54	0.71	1.48	0.74
Glucose	571.00	6.48	5.99	3.42	17.54	5.23	7.13	1.87
BMI	571.00	25.20	24.68	13.13	43.21	22.60	27.74	3.91

Supplementary Table 6 Correlation between demographic, biochemical and clinical parameters among males

	Age	TSH	Glucose	BMI
Age	1.0000	**-0.1210*§**	**0.1434*§**	**-0.1159*§**
TSH	**-0.1210*§**	1.0000	**-0.1506*§**	0.0750
Glucose	**0.1434*§**	**-0.1506*§**	1.0000	0.0682
BMI	**-0.1159*§**	0.0750	0.0682	1.0000

**P* < 0.05 based on Spearman rank-order correlations
§ denote significance after Bonferroni adjustment

Supplementary Table 7 Descriptive statistics of demographic, biochemical and clinical variables in the ALS cohort

	Valid N	Mean	Median	Minimum	Maximum	Lower Quartile	Upper Quartile	Std.Dev.
Age	340.00	62.58	63.23	25.14	85.98	55.20	70.27	10.95
TSH	340.00	1.17	1.02	0.04	9.54	0.69	1.43	0.79
Glucose	340.00	6.56	6.16	3.42	17.54	5.29	7.25	1.90
pNfH_CSF	340.00	2,467.69	1,740.00	188.00	16,459.00	784.50	3,441.50	2,417.91
BMI	340.00	25.36	24.69	14.79	43.21	22.39	27.96	4.26

Supplementary Table 8 Descriptive statistics of demographic, biochemical and clinical variables in the ALS cohort (females)

	Valid N	Mean	Median	Minimum	Maximum	Lower Quartile	Upper Quartile	Std.Dev.
Age	118.00	62.75	63.09	25.14	83.95	55.45	70.89	10.87
TSH	118.00	1.13	1.02	0.08	3.44	0.72	1.38	0.63
Glucose	118.00	6.53	6.19	3.55	15.55	5.29	7.23	1.90
pNfH_CSF	118.00	2,338.31	1,690.50	188.00	10,078.00	811.00	3,230.00	2,195.99
BMI	118.00	24.84	24.23	15.06	41.51	21.63	27.64	4.49

Supplementary Table 9 Correlations between demographic, biochemical and clinical parameters among females

	Age	TSH	Glucose	pNfH_CSF	BMI
Age	1.0000	-0.0977	0.1560	**-0.0728**	-0.0001
TSH	-0.0977	1.0000	0.0355	**0.1640**	-0.0526
Glucose	0.1560	0.0355	1.0000	**0.0001**	0.1010
pNfH_CSF	-0.0728	0.1640	0.0001	**1.0000**	-0.1026
BMI	-0.0001	-0.0526	0.1010	**-0.1026**	1.0000

Supplementary Table 10 Descriptive statistics of demographic, biochemical and clinical variables in the ALS cohort (males)

	Valid N	Mean	Median	Minimum	Maximum	Lower Quartile	Upper Quartile	Std.Dev.
Age	222.00	62.49	63.23	30.99	85.98	55.05	70.06	11.01
TSH	222.00	1.19	1.02	0.04	9.54	0.67	1.47	0.87
Glucose	222.00	6.58	6.16	3.42	17.54	5.29	7.39	1.90
pNfH_CSF	222.00	2,536.47	1,834.50	188.00	16,459.00	740.00	3,501.00	2,530.05
BMI	222.00	25.63	24.95	14.79	43.21	22.65	28.29	4.12

Supplementary Table 11 Correlations between demographic, biochemical and clinical parameters among males

	Age	TSH	Glucose	pNfH_CSF	BMI
Age	1.0000	-0.1113	**0.2902*§**	**-0.1185**	-0.0887
TSH	-0.1113	1.0000	**-0.1955*§**	**0.0436**	0.0364
Glucose	**0.2902*§**	**-0.1955*§**	1.0000	**-0.0389**	0.0981
pNfH_CSF	-0.1185	0.0436	-0.0389	**1.0000**	-0.0759
BMI	-0.0887	0.0364	0.0981	**-0.0759**	1.0000

**P* < 0.05 based on Spearman rank-order correlations
§ denote significance after Bonferroni adjustment

Supplementary Table 12 Descriptive statistics of demographic, biochemical and clinical parameters of patients without L-Thyroxine substitution therapy

	Valid N	Mean	Median	Minimum	Maximum	Lower Quartile	Upper Quartile	Std.Dev.
Age	831.00	62.80	63.47	19.21	87.84	55.38	71.76	11.55
TSH	831.00	1.18	1.02	0.00	7.96	0.70	1.45	0.80
Glucose	831.00	6.53	5.99	3.26	18.11	5.23	7.31	1.96
BMI	831.00	25.02	24.49	13.13	44.96	22.28	27.55	4.21
pNfH_CSF	302.00	2,451.12	1748.00	188.00	16,459.00	811.00	3,363.00	2,387.88

Supplementary Table 13 Descriptive statistics of demographic, biochemical and clinical parameters of female patients without L-Thyroxine substitution therapy

	Valid N	Mean	Median	Minimum	Maximum	Lower Quartile	Upper Quartile	Std.Dev.
Age	305.00	62.80	62.93	24.64	87.84	55.62	71.32	11.19
TSH	305.00	1.11	1.01	0.00	5.42	0.65	1.44	0.67
Glucose	305.00	6.55	6.04	3.51	17.54	5.17	7.37	2.13
BMI	305.00	24.77	24.13	14.27	44.96	21.37	27.43	4.81
pNfH_CSF	105.00	2,323.70	1,682.00	188.00	16,459.00	725.00	3,251.00	2,325.36

Supplementary Table 14 Descriptive statistics of demographic, biochemical and clinical parameters of male patients without L-Thyroxine substitution therapy

	Valid N	Mean	Median	Minimum	Maximum	Lower Quartile	Upper Quartile	Std.Dev.
Age	526.00	62.81	63.60	19.21	85.98	55.34	71.79	11.77
TSH	526.00	1.21	1.04	0.00	7.96	0.73	1.46	0.86
Glucose	526.00	6.51	5.96	3.26	18.11	5.28	7.26	1.87
BMI	526.00	25.17	24.69	13.13	37.87	22.65	27.68	3.82
pNfH_CSF	197.00	2,519.04	1,754.00	188.00	13,102.00	879.00	3,449.00	2,423.65

Supplementary Figures

Supplementary Figure 1 Scatterplot illustrating the relationship between TSH and age

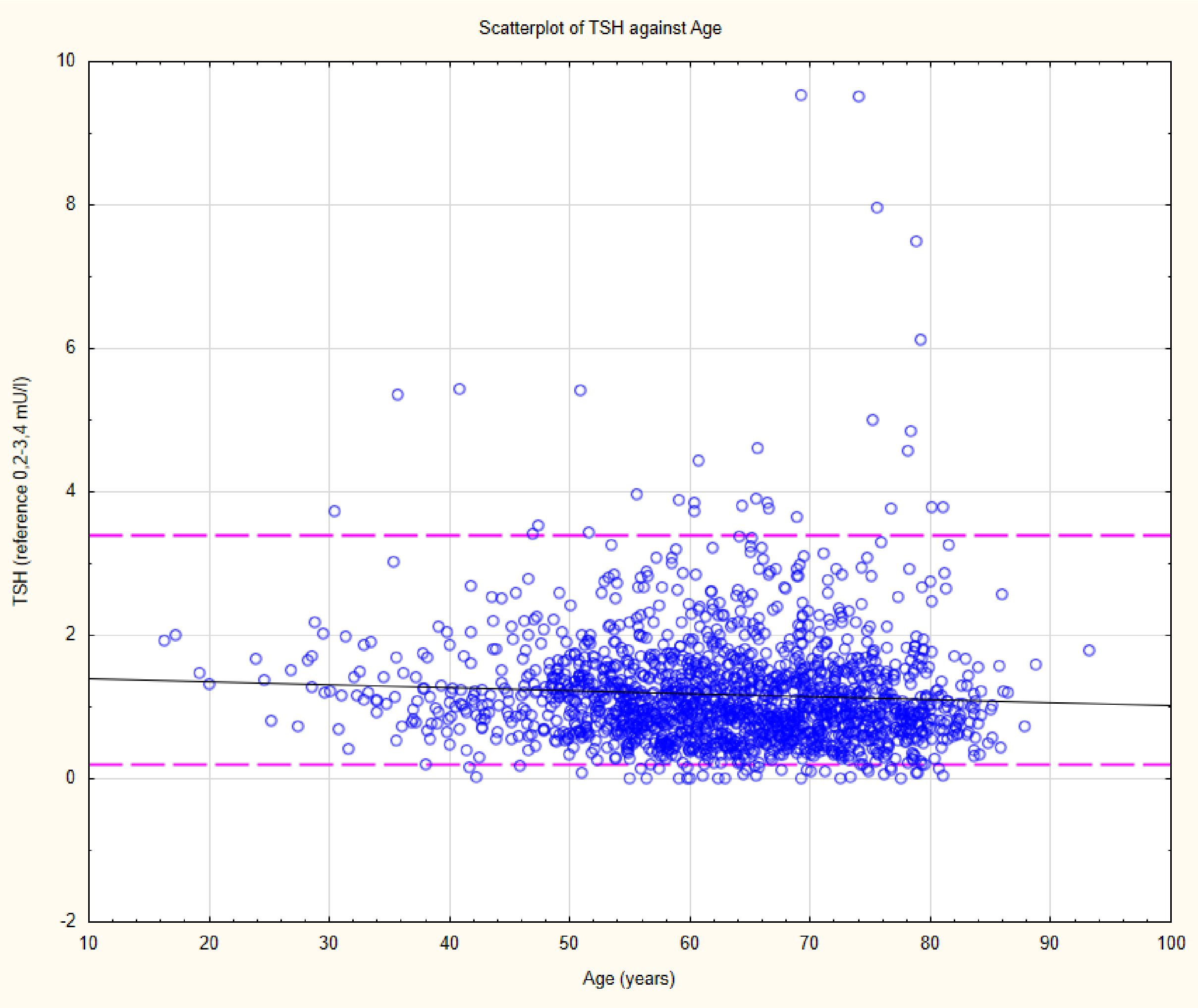

Spearman's rank-order correlation reveals weak negative correlation between TSH and age even after Bonferroni adjustment (r = –0.0920, $P < 0.05$, n = 1754). Each dot represents individual patient.

Alt text: Scatterplot showing age-related changes in TSH levels, featuring reference limits and the median trajectory.

Abbreviations: TSH – thyroid-stimulating hormone.

Supplementary Figure 2 Scatterplot illustrating the relationship between BMI and age

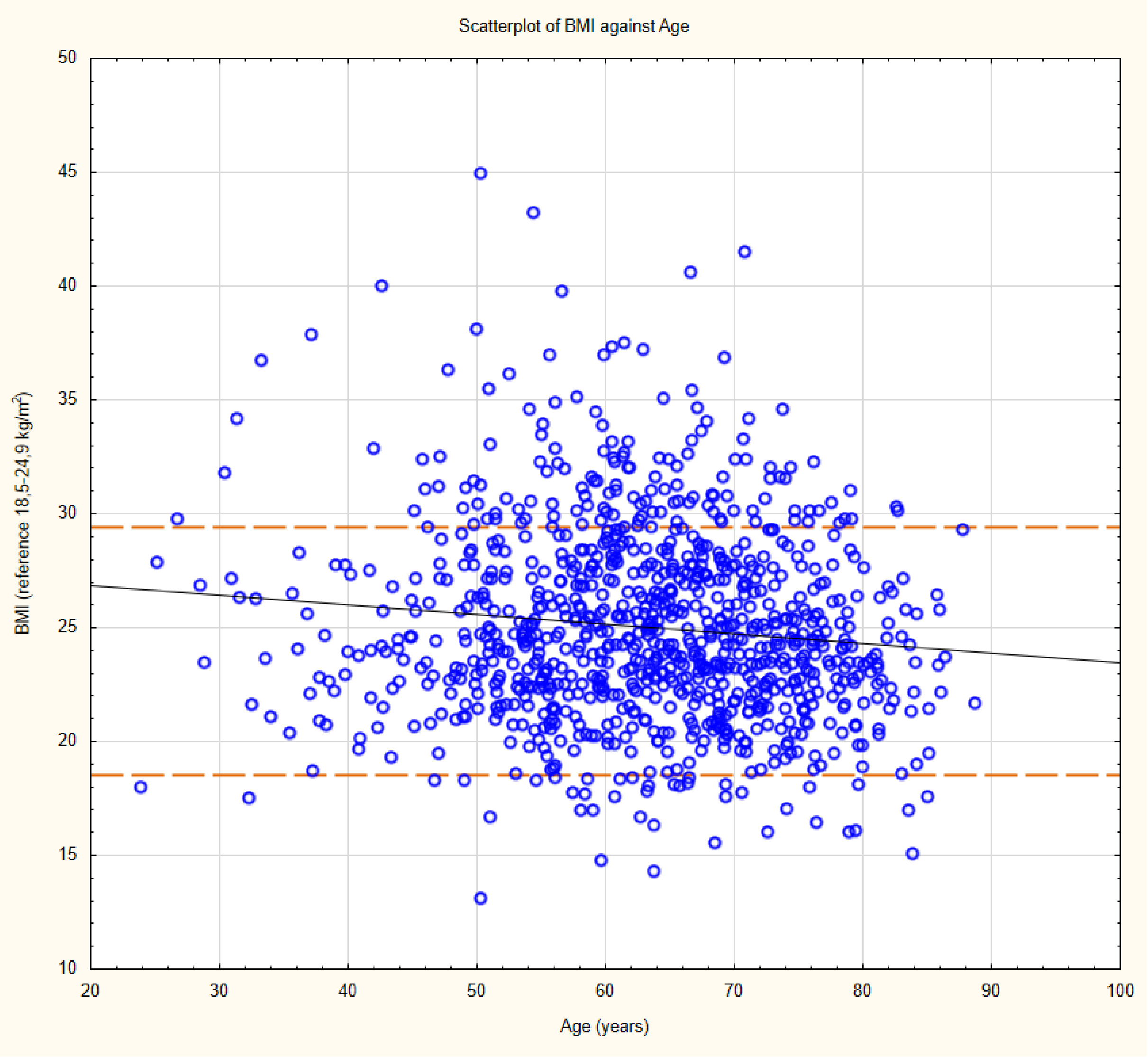

Spearman's rank-order correlation reveals significant negative correlation between BMI and age even after Bonferroni adjustment (r = –0.1017, $P < 0.05$, n = 940). Each dot represents individual patient.

Alt text: Scatterplot showing age-related changes in BMI, featuring reference limits and the median trajectory.

Abbreviations: BMI – body mass index.

Supplementary Figure 3 Scatterplot illustrating the relationship between blood glucose and age

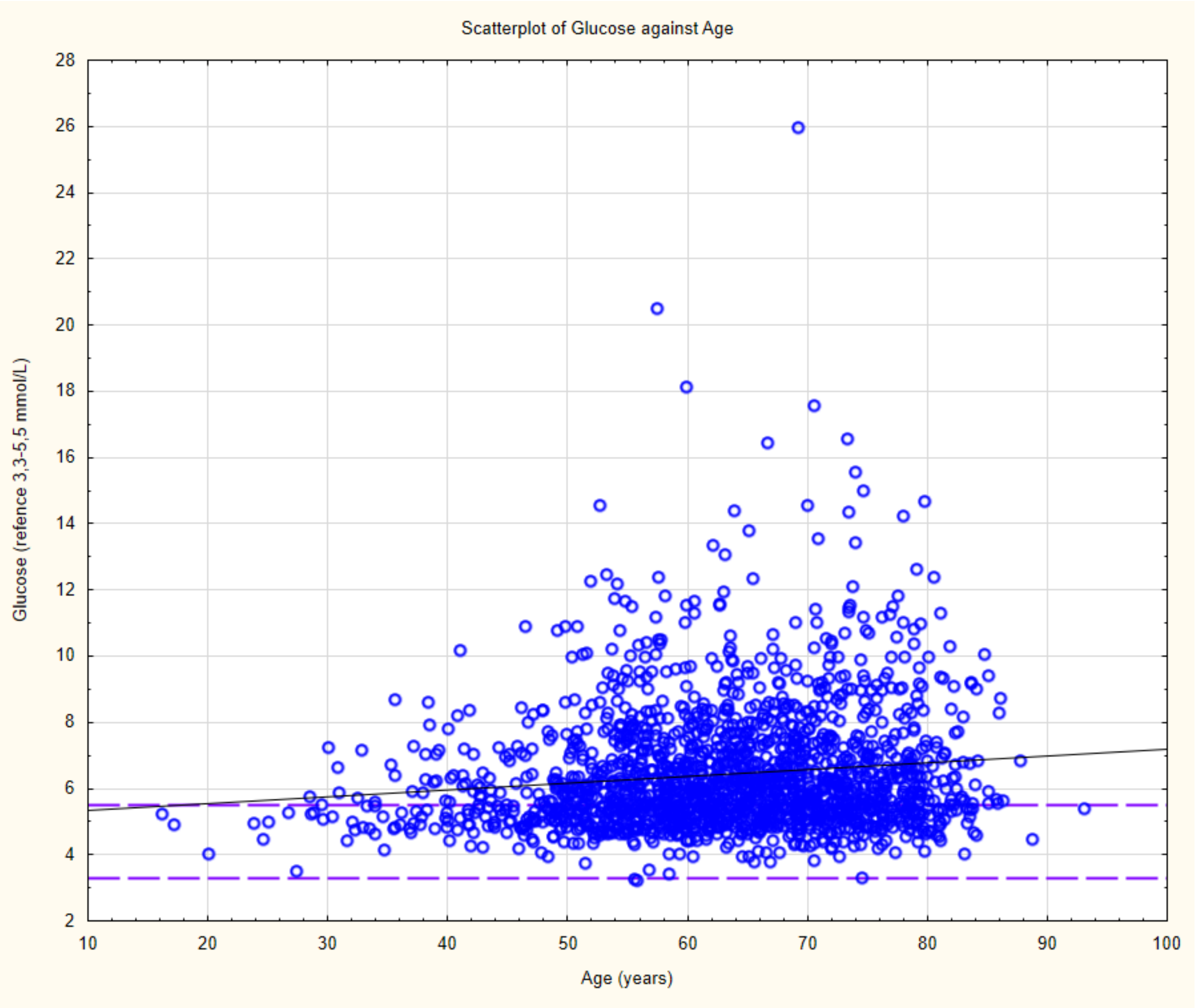

Spearman's rank-order correlation reveals significant positive correlation between glucose and age even after Bonferroni adjustment ($r = 0.1501$, $P < 0.05$, $n = 940$). Each dot represents individual patient.

Alt text: Scatterplot showing age-related changes in glucose levels, featuring reference limits and the median trajectory.

Supplementary Figure 4 Scatterplot illustrating the relationship between TSH and glucose

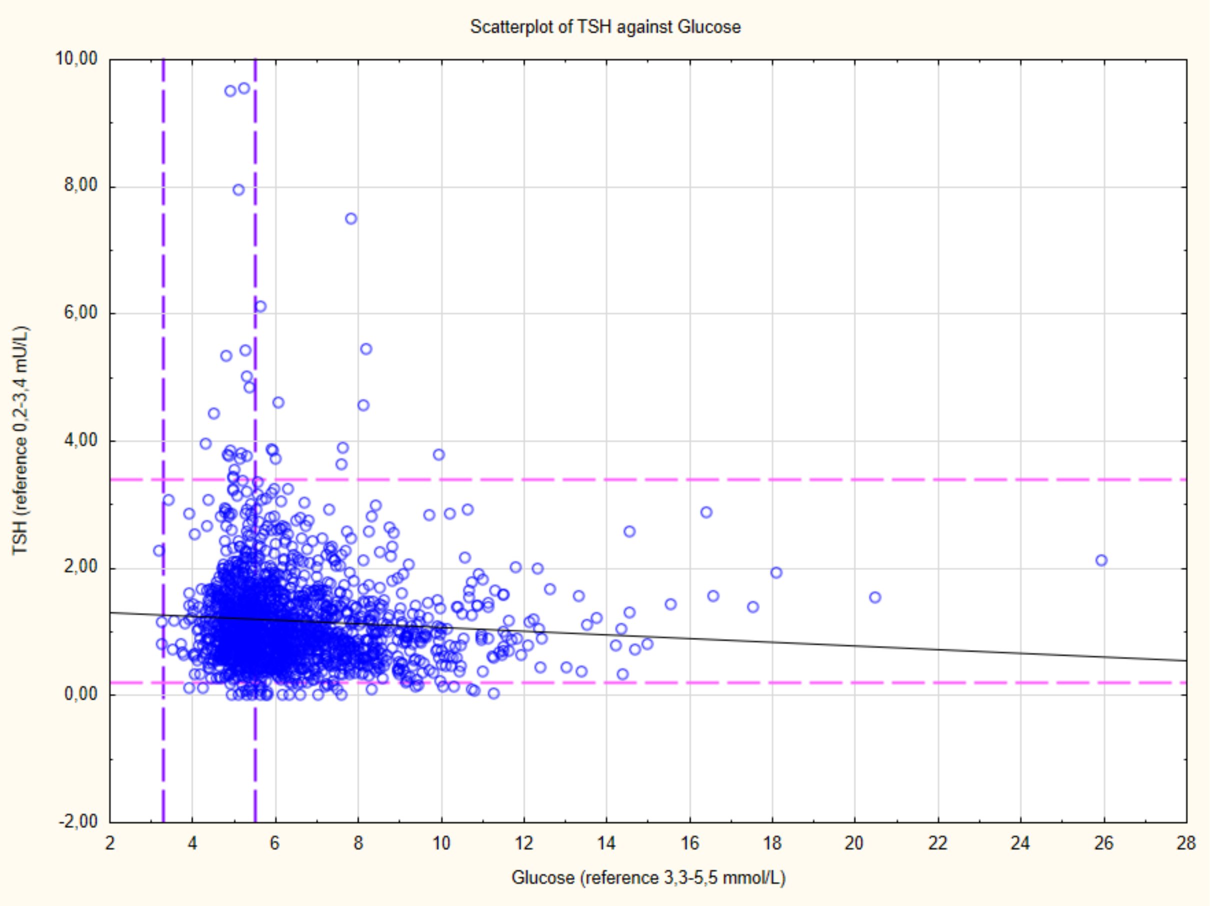

Spearman's rank-order correlation reveals significant negative correlation between TSH and glucose even after Bonferroni adjustment *(r = –0.1051, $P < 0.05$, n = 940)*. Each dot represents individual patient.

Alt text: Scatterplot showing association between TSH and glucose levels, featuring reference limits and the median trajectory.

Abbreviations: TSH – thyroid-stimulating hormone.

Supplementary Figure 5 Scatterplot illustrating the relationship between BMI and glucose

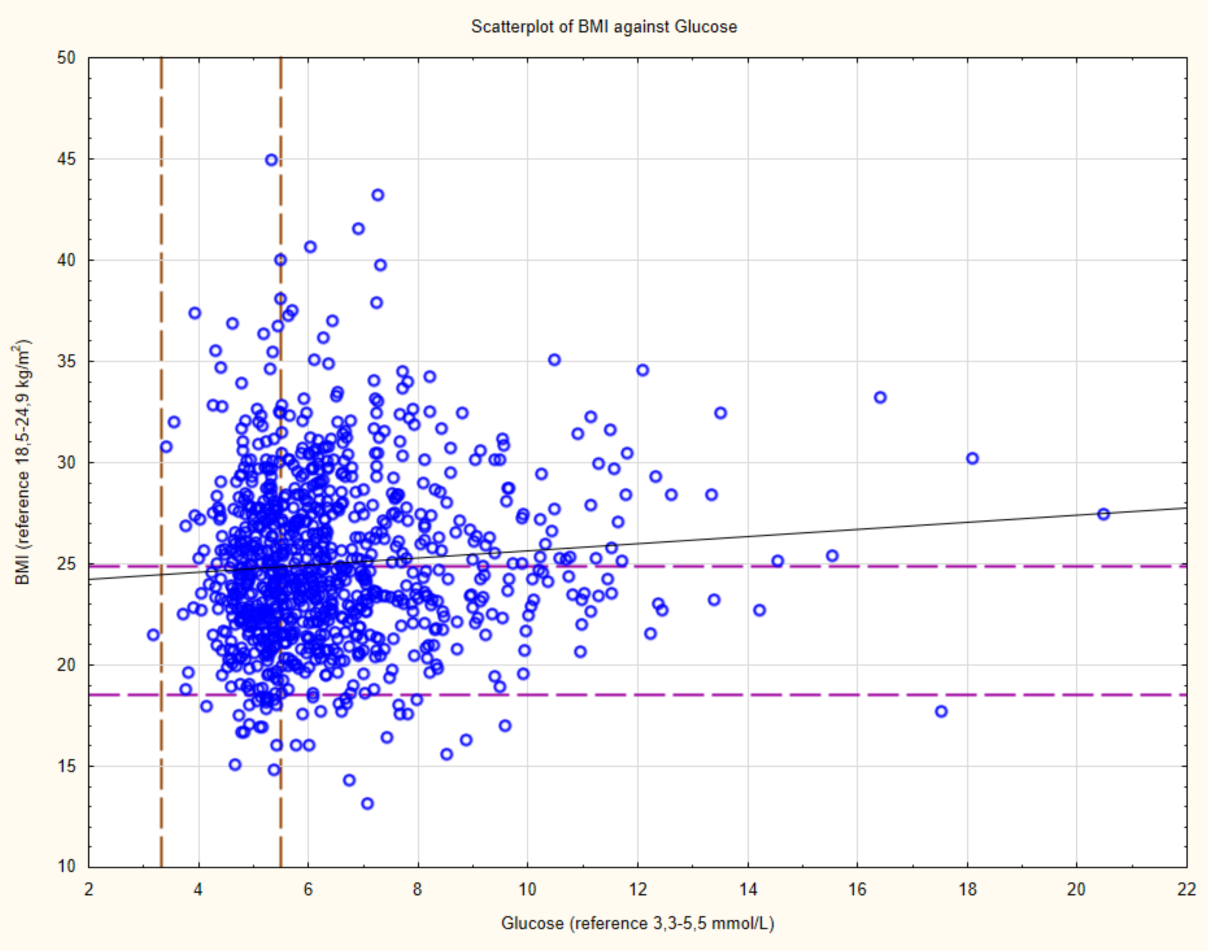

Spearman's rank-order correlation reveals weak positive correlation between BMI and glucose even after Bonferroni adjustment ($r = 0.0869$, $P < 0.05$, $n = 940$). Each dot represents individual patient.

Alt text: Scatterplot showing association between BMI and glucose levels, featuring reference limits and the median trajectory.

Abbreviations: BMI – body mass index.

Supplementary Figure 6 Scatterplot illustrating the relationship between glucose and age in female patients

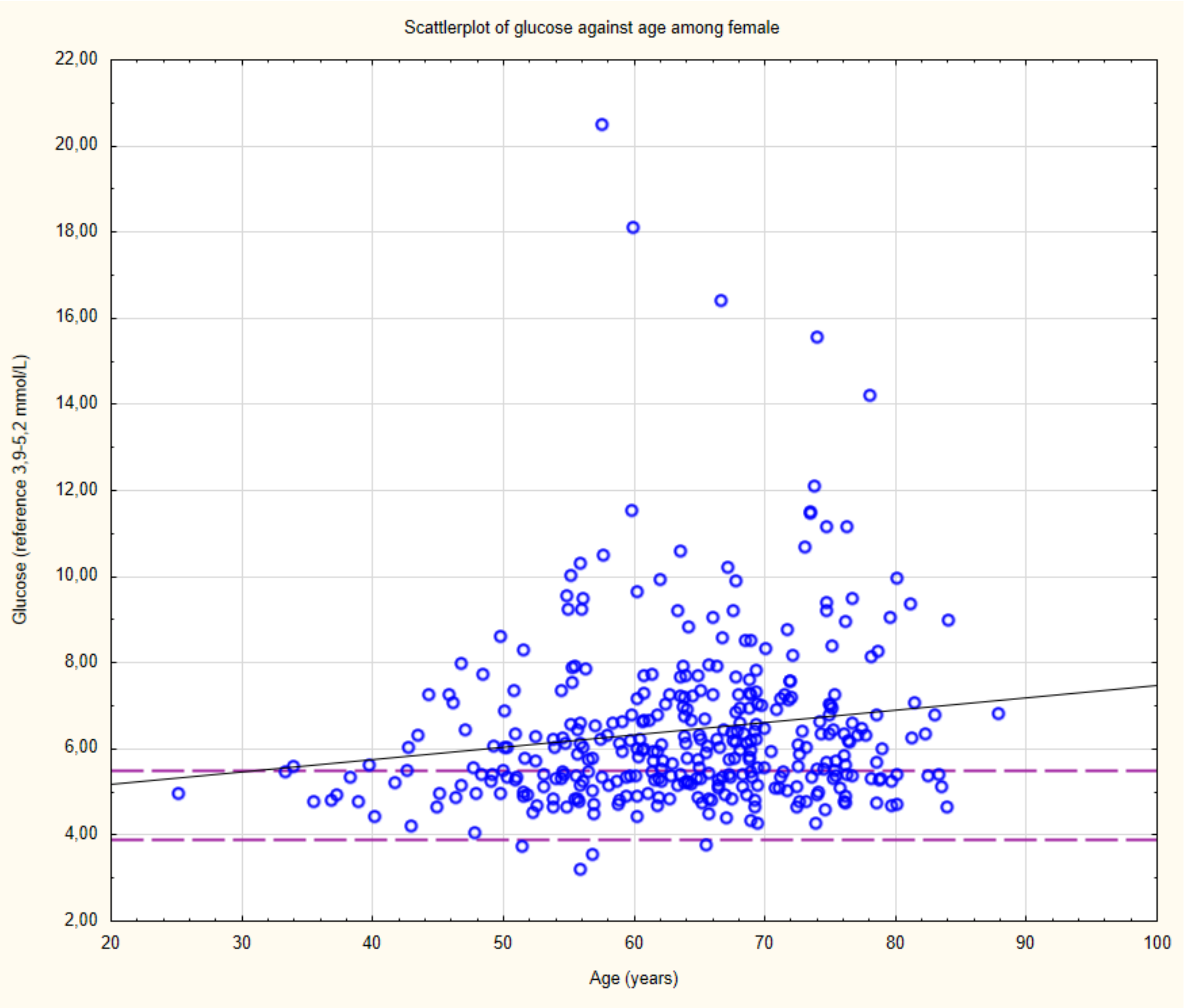

Spearman's rank-order correlation reveals significant positive correlation between glucose and age even after Bonferroni adjustment ($r = 0.1649$, $P < 0.05$, $n = 369$). Each dot represents individual patient.

Alt text: Scatterplot showing age-related changes in glucose levels in female patients, featuring reference limits and the median trajectory.

Supplementary Figure 7 Scatterplot illustrating the relationship between TSH and age in male patients

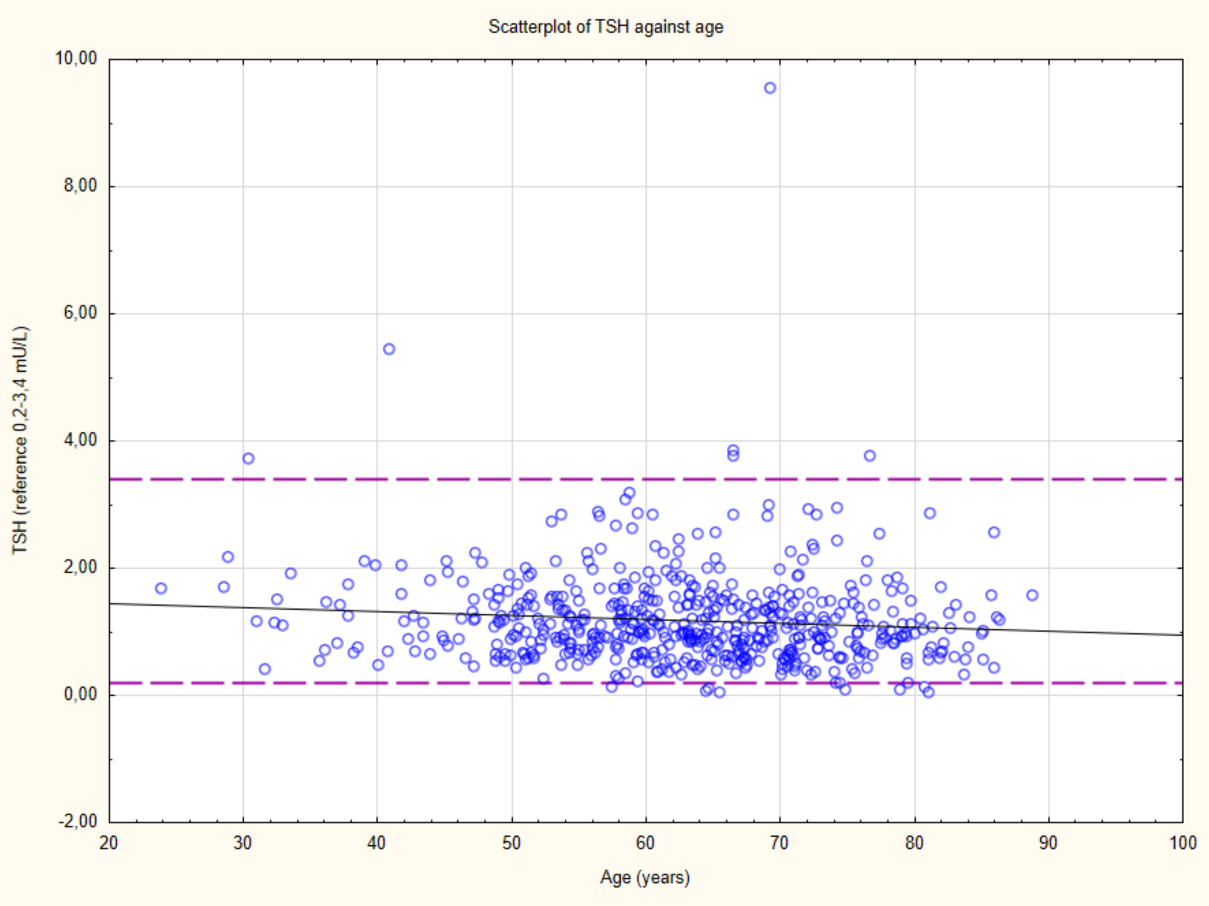

Spearman's rank-order correlation reveals significant negative correlation between TSH and age even after Bonferroni adjustment *(r* = –0.1210, *P* < 0.05, *n* = 571). Each dot represents individual patient.

Alt text: Scatterplot showing age-related changes in TSH in male patients, featuring reference limits and the median trajectory.

Abbreviations: TSH – thyroid-stimulating hormone.

Supplementary Figure 8 Scatterplot illustrating the relationship between BMI and age in male patients

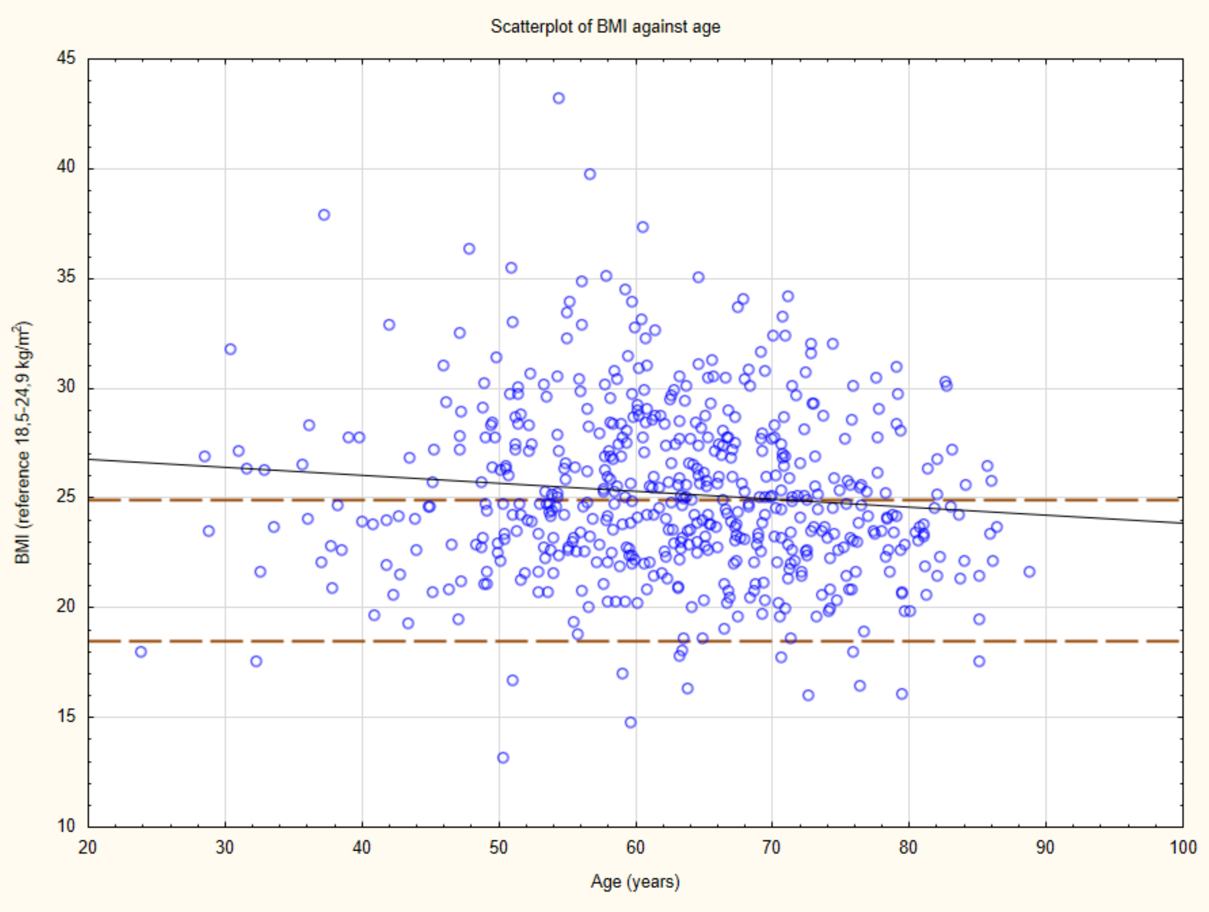

Spearman's rank-order correlation reveals significant negative correlation between BMI and age even after Bonferroni adjustment (r = –0.1159, $P < 0.05$, n = 571). Each dot represents individual patient.

Alt text: Scatterplot showing age-related changes in BMI in male patients, featuring reference limits and the median trajectory.

Abbreviations: BMI – body mass index.

Supplementary Figure 9 Scatterplot illustrating the relationship between glucose and age in male patients

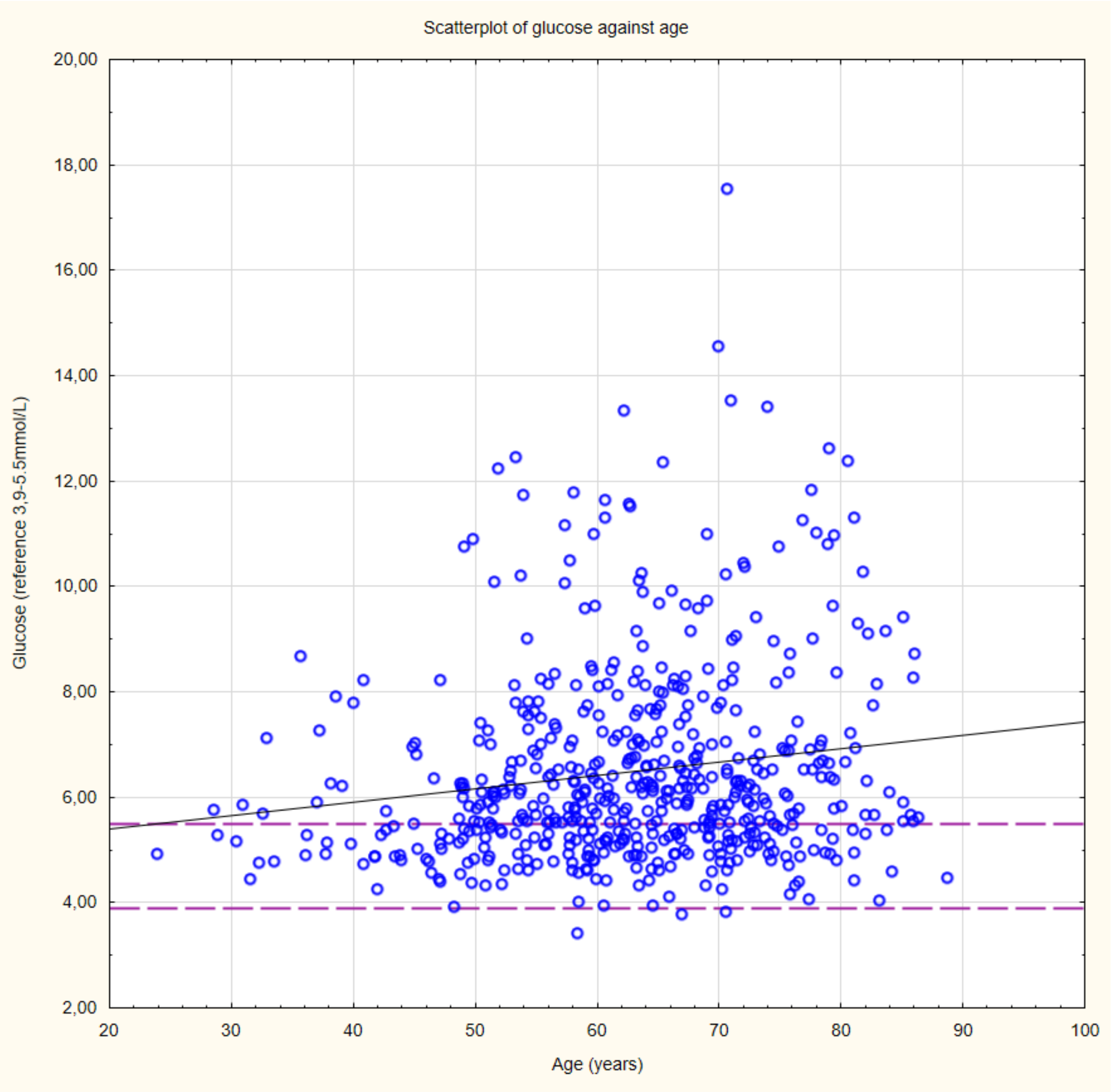

Spearman's rank-order correlation reveals significant positive correlation between glucose and age even after Bonferroni adjustment ($r = 0.1434$, $P < 0.05$, $n = 571$). Each dot represents individual patient.

Alt text: Scatterplot showing age-related changes in glucose levels in male patients, featuring reference limits and the median trajectory.

Supplementary Figure 10 Scatterplot illustrating the relationship between TSH and glucose in male patients

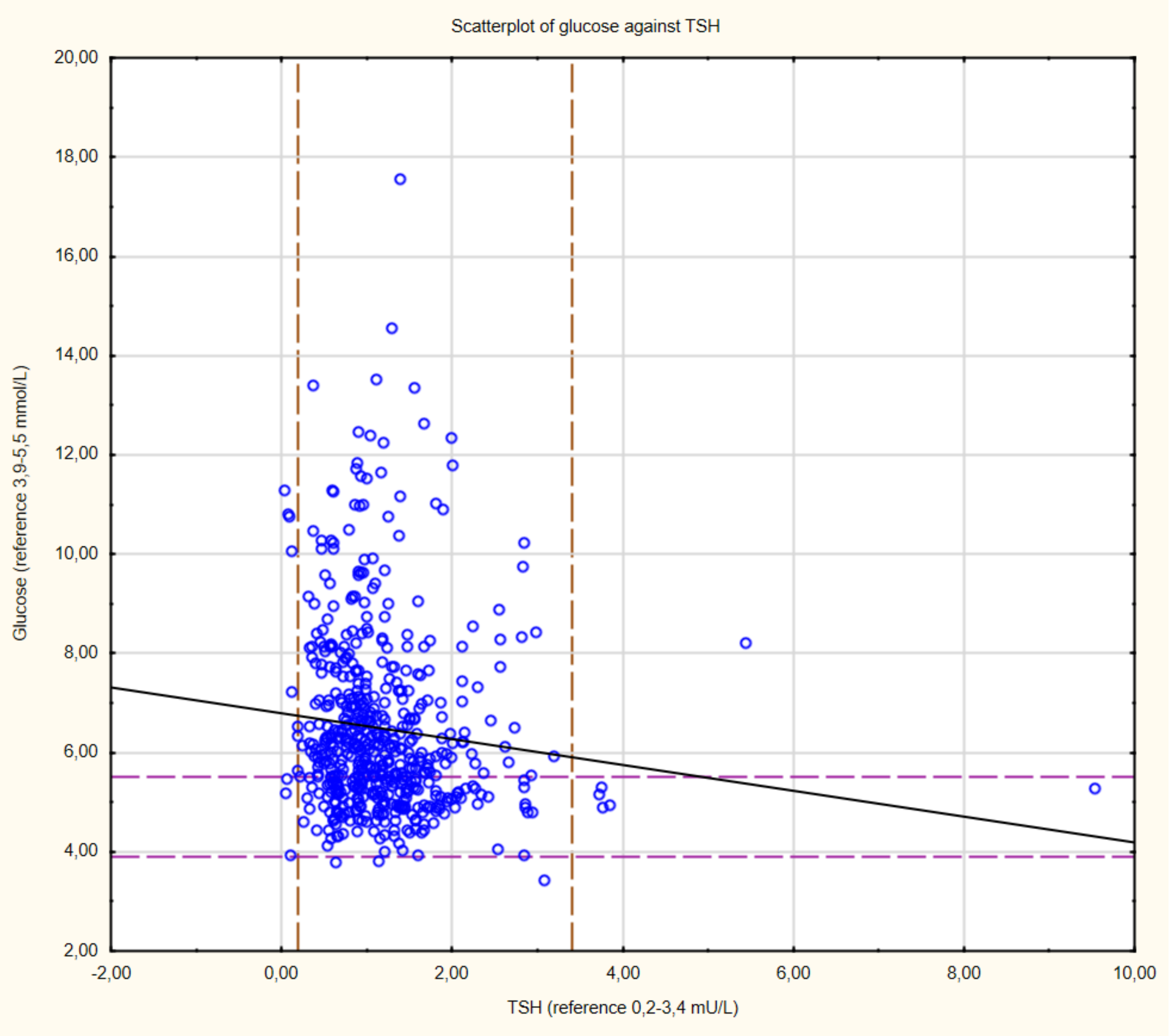

Spearman's rank-order correlation reveals significant negative correlation between glucose and TSH even after Bonferroni adjustment ($r = -0.1506$, $P < 0.05$, $n = 571$). Each dot represents individual patient.

Alt text: Scatterplot showing association between glucose and TSH levels in male patients, featuring reference limits and the median trajectory.

Abbreviations: TSH – thyroid-stimulating hormone.
